# Examining the Growing Demand for Surgical Care in Rural Communities and Novel Approaches to Achieving a Sustainable Surgical Workforce: A Narrative Review

**DOI:** 10.7759/cureus.43817

**Published:** 2023-08-20

**Authors:** Brittany A Long, Michael J Sweeney

**Affiliations:** 1 General Surgery, Florida State University College of Medicine, Tallahassee, USA

**Keywords:** rural population, fellowship, physician shortage, surgical workforce, rural surgeon, rural surgery, surgeon shortage, surgery residency, surgical education, surgery

## Abstract

Surgery continues to be an increasingly vital component of public health and aspect of patient care in rural communities. An anticipated shortage of surgeons within the next decade in the United States prompts a growing concern for increasing the delivery of essential surgical care to these populations. When considering the existing barriers to surgical healthcare in rural communities, there is a sense of urgency to identify innovative approaches that will promote a sustainable surgeon workforce. A narrative review was conducted to investigate the current state of access to essential surgical care in rural communities. Qualitative and quantitative data were collected to better understand the key issues in rural healthcare and to provide statistical data related to the status of the surgical workforce. With the anticipated shortage of surgeons in both rural and urban areas, this review highlights the importance of enacting immediate measures to address the concern. This review has accomplished the initial objectives of gaining a better understanding of the current state of access to surgical care in rural communities and utilizing this knowledge to provide recommendations to readily attain a sustainable number of rural surgeons. With each approach addressing ways to address the contributory issues to the surgeon shortage, this review reveals a new avenue of integrating valuable aspects from each approach, rather than relying on a single approach. In particular, enhancing the overall pipeline of medical training to attending status may prove to be more beneficial for achieving this goal. Ultimately, this may be accomplished by introducing additional rural surgical mentorship opportunities for medical students, developing a rural surgery fellowship, and incorporating a market-based response that will correspond to attractive incentives that help to retain a sustainable number of surgeons working in rural areas.

## Introduction and background

Access to healthcare continues to be an ongoing public health concern. Several approaches have been taken to address the healthcare needs of rural, minority, and underserved communities. However, there is additional work that must be done to continue bridging the gap between access to sustainable healthcare and the barriers that these populations face. While disparities in access to primary healthcare present a key public health issue, surgical care has become an increasingly vital component of public health that must be considered [1]. It is predicted that there will be approximately 30,760 practicing general surgeons with a demand for 33,730 surgeons by 2025 [2]. In rural communities specifically, where there are currently only 4.67 surgeons per 100,000 people, several factors contribute to the challenges these patients face regarding access to essential and routine surgeries [3-4]. In addition to existing health disparities, the declining number of surgeons is of particular concern as it significantly impacts both rural and metropolitan areas [3]. By 2032, it is predicted that there will be an estimated shortage of approximately 23,000 surgeons in the United States [5]. Studies have suggested that this trend is likely due to a multitude of factors, including, but not limited to, physician burnout, an aging surgeon workforce, and a stagnant number of newly trained surgeons [6-7]. While general surgeons serve a crucial role in delivering care to rural populations through both routine and emergent procedures, a sustained number of surgeons choosing to practice in rural areas has not yet been achieved [3,8]. 

With the supply of surgeons predicted not to meet patient demand, there is a sense of urgency to identify key approaches to ensure a sustainable future of surgical healthcare. Even more concerning are the implications this will have on the aging and elder populations in rural communities, who face complex health needs that may warrant surgical care [5]. This research aims to examine the specific aspect of access to essential surgical care in rural populations in efforts to find novel approaches to address the anticipated shortage of rural surgeons.

The following objectives and questions were used to guide the review: 

Objective 1: Examine the current state of access to essential surgical care in rural communities.

Question 1: What components of surgical care and critical procedures are most commonly needed in rural populations?

Question 2: What are the factors that contribute to the challenges rural populations face regarding access to essential surgical care?

Objective 2: Develop novel recommendations to bridge the gap between demand for essential surgical care and the limited supply of surgeons in rural areas.

Question 1: How is the predicted number of practicing surgeons going to compare to the demand in surgical needs for rural populations within the next 10 years?

Question 2: What are the factors that can promote initiatives to increase the number of surgical providers in rural populations?

## Review

**Figure 1:**
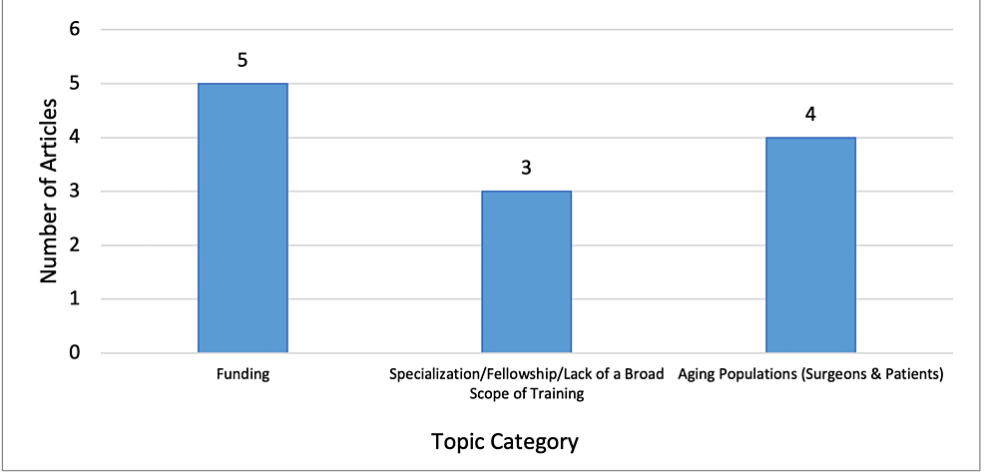
Article distribution related to "contributory issues" to surgeon shortage

**Figure 2:**
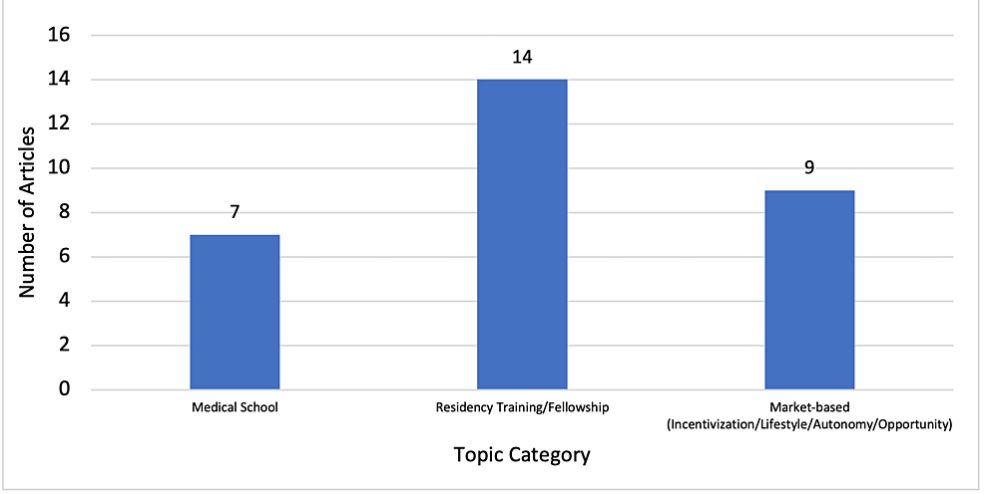
Article distribution related to "solutions" to surgeon shortage

A literature review was conducted using two databases: PubMed and EMBase. The following keywords were used in all database searches: “rural surgery,” “rural surgeon,” and “surgeon shortage.” Using advanced search settings, the publication years 2009-2021 were selected with articles ordered according to relevance. Inclusion criteria included the following: peer-reviewed articles, articles published between 2009 and 2021, articles related to rural surgery in the United States, articles related to general surgery, and articles in the English language. A total of 35 articles were selected for review. As a part of the literature review, issues contributing to the shortage of surgeons in rural areas were also examined to better understand the implications of changes needed to achieve a sustainable surgical workforce. The issues identified through the literature review, as summarized by Figure 1, can be categorized into the following topics: 1) funding, 2) fellowship/lack of a broad scope of training, and 3) aging populations (surgeons and patients). Similarly, existing solutions that have been proposed can be categorized into the following types of approaches: 1) medical school, 2) residency training/fellowship, and 3) market-based approaches (incentivization/lifestyle/autonomy/opportunity), as shown in Figure 2. 

Issues contributing to surgeon shortage

Funding 

With the anticipated surgeon shortage in rural areas, it is important to consider the influence of funding on the issue at hand. The Balanced Budget Act (BBA) of 1997 introduced a cap on federally funded graduate medical education (GME) [9]. Since the passage of the BBA, the number of all types of residences has remained stagnant [9]. This in turn has led to a limited number of residency positions available to graduating medical students, despite the increase in medical student enrollment each year. For general surgery specifically, the number of categorical residency positions has stayed the same with a range of 900-1,200 since the introduction of the BBA [9]. To demonstrate this idea further, even in 2017, there were 43,157 applicants for 27,860 first-year residency positions in Accreditation Council for Graduate Medical Education (ACGME)-accredited programs [10]. Interestingly, one study illustrated that over 150 hospitals could readily accommodate new general surgery residency programs, but this is not likely to happen due to a lack of federal funding for these residency positions [10]. Despite the fact that rural residents comprise 12% of the 35 million hospitalizations in the United States as of 2010, there is a concern for the increase in closures of rural care hospitals, with many categorized as critical access hospitals (CAHs) [11]. In 1997, the BBA introduced the designation of CAHs to support rural populations in need of healthcare and emergency services [12]. Since 2005, there have been 170 rural hospital closures [12]. This trend is likely to be due to several factors, including low patient occupancy rates and limited financial resources [11]. After all, the “rural surgery triad” as described by Nakayama et al. includes the interdependent relationship among the surgeon, the community he or she serves, and the hospital he or she practices [13]. This triad is crucial in understanding how surgeons can impact hospital finances, especially in rural areas. Of note, rural hospitals are aware of the significance of surgical services for these populations, but hospitals often face difficulties recruiting and retaining surgeons, in addition to the lack of funding available [12].

Specialization/Fellowship and Lack of a Broad Scope of Training 

An increasing number of residents pursuing fellowship training following general surgery residency highlights the concern for attaining a sustainable rural surgeon workforce. In fact, nearly 85% of graduating surgical residents are choosing to pursue a fellowship [14]. In a 2020 study, survey results obtained from surgical residents in a hybrid community/academic program revealed that the confidence level in independently performing cases was a driving factor for pursuing a specialized fellowship in 55% of residents [14]. This study suggests that introducing earlier autonomy to residents may aid in producing more confident surgeons upon completion of residency training, minimizing the growing gap between the supply of rural general surgeons and specialists. Other concerns for the increase in specialized fellowship training include graduating residents’ perceived need for attaining a “competitive edge” in the surgical marketplace [15]. A recent study has supported the idea that over half of graduating general surgery residents perceive specialty training as being essential to having a successful career [16]. Thus, by introducing a more robust and broad scope of training in procedures beyond general surgery, surgeons may find themselves better positioned to pursue a career in rural general surgery.

Aging Populations (Surgeons and Patients) 

With the continued growth in the elderly population, the general surgeon to population ratio remains of imminent concern [4]. When considering the growing population of aging individuals, including those who are practicing general surgeons, the anticipated shortage of rural general surgeons is particularly alarming. As a 2020 study illustrates, the rural general surgeon is characteristically male and aged 50-55 years old, with approximately 60% of practicing rural general surgeons planning to retire within the next 10 years [2]. When compared to the older generations of general surgeons who have been more broadly trained, the most recently trained general surgeons are not trained in specialties, such as obstetrics/gynecology or orthopedics [17]. The shortage of general surgeons is further compounded by the increase in the proportion of elderly patients in need of surgical care, specifically when considering the baby-boom population that will be entering its geriatric years [17]. In elderly adults, common procedures include those performed in the fields of orthopedic surgery and cardiovascular surgery [18]. However, in a study examining age-specific rates of surgical use by Etzioni et al., there was an anticipated 31% increase in general surgery cases by 2020 [18]. With the anticipated shortage of trainees in nearly all surgical specialties, it may be beneficial to expand the breadth of training for the future rural general surgeon to accommodate the needs of the aging patient populations.

Solutions to obtaining a more sustainable surgical workforce 

Medical School

Colleges and medical schools serve as a valuable time for students to explore various specialties prior to applying to residency programs, suggesting an opportunity to recruit students to rural general surgery specifically. Many medical schools have made efforts to recruit students who demonstrate an increased likelihood of practicing in rural primary care, but it may be beneficial to enact similar efforts to recruit rural general surgeons [19]. In a study of four Wisconsin surgical training programs, factors contributing to the selection of rural surgical training included the following: attending a nonurban high school or college (P < 0.001), having a spouse/partner who grew up in a nonurban area (P < 0.022), and interest in hunting birds (P < 0.010) or large game (P < 0.001) [20]. Other studies have suggested that the rural surgeon pipeline must be more streamlined. As Henry et al. summarized, there is a need to accomplish the following: (1) establish structured contact between rural high school students and the medical profession; (2) increase selection of rural students into medical schools; (3) provide rural exposure during medical training; (4) increase selection of rural students into surgical training; (4) provide rural tracts in surgical training; and (5) enact a concerted effort to attract and retain rural general surgeons. [21].

Rural general surgeon mentorship may also provide an avenue for recruiting and retaining individuals who will practice in a rural area following residency training [22]. A lack of exposure to rural surgery can contribute to the shortage of the rural general surgeon workforce as graduating medical students are less likely to seek a position in this area [23]. Fortunately, special interest groups established during medical school have often been cited as a means of increasing awareness of a particular specialty for medical students [24]. These groups usually share the common goal of fostering mentorship among students, faculty, and residents in the specialty of interest, thereby introducing the opportunity to increase exposure to rural surgery as a specialty. Furthermore, by promoting the positive attributes of being a rural surgeon through opportunities for shadowing or rotating alongside the surgeon throughout medical school, awareness and exposure to rural life could be achieved earlier on in medical training [22]. Along with mentorship, there is strong support for recruiting medical students who grew up in a rural area. In a 2021 study by Frohne et al., the primary reason for selecting rural practice locations was largely due to previous exposure to rural areas while growing up as evidenced by 69% of survey respondents consisting of attending surgeons, residents, and fellows [22]. As summarized by the concept of nature versus nurture of rural practice presented by Mccarthy et al., the “nurture” aspects of training during medical school and residency, including curricula, faculty, or rotations, may provide opportunities to increase awareness and interest in rural practice [20].

Residency Training 

Several articles have highlighted the need for a broader scope of training during general surgery residency. Meanwhile, it is estimated that approximately 10% of US general surgery residency programs meet one out of three of the following criteria, namely, rural location, rural-focused curriculum, or self-identified interest in rural training; when evaluating the likelihood of preparing rural surgeons, there is still a need to establish a coordinated effort that will foster more graduating surgeons who are willing to practice rurally [25]. Generally, rural surgeons typically perform an overall higher number of procedures in addition to more subspecialty and endoscopic procedures as compared to urban surgeons [26,27]. In a 2012 study examining the adequacy of preparation of general surgery training for rural surgery practice based in North and South Dakota, it was reported that 5,666 of 46,052 procedures in rural practice (12.3%) were specialty procedures [28]. These included procedures performed in specialties, such as obstetrics/gynecology, vascular, orthopedics, cardiothoracic surgery, urology, and otolaryngology. A similar study published in 2019 based in Kansas demonstrated the significant percentage of rural general surgeons performing specialist procedures, including the following: hysterectomies (51.2%), thyroidectomies (81.4%), parathyroidectomies (60.5%), carotid endarterectomies (11.6%), video-assisted thoracoscopic surgery (37.2%), and lobectomies (23.3%) [26]. To further support this idea, a study examining the role of a rural surgical rotation during residency in graduating residents from a University of Tennessee-Knoxville residency program revealed that the three-month rural rotation involved a higher caseload (93 cases per resident on urban rotation vs. 164 cases per resident on rural rotation), with a significantly higher number of procedures in endoscopy, gynecology, urology, thoracic surgery, and vascular surgery [29]. These data readily demonstrate the need for a broader scope of surgical training in efforts to instill a greater sense of preparedness and competency for the future rural general surgeons. 

An additional survey completed by practicing rural surgeons in 2018 highlights the necessary priorities in training the rural general surgeon to include enhancing skills in advanced laparoscopy, endoscopy, and basic non-general surgery subspecialty procedures [30]. Three additional articles support these findings in providing general surgeons robust training and experience to perform a broad scope of procedures [31,32,33]. Rossi et al. described the need for broad training in specialty procedures not previously discussed, to include plastic surgery (e.g., skin grafts, common flaps, and tissue expanders for breast reconstruction), interventional radiology (US-guided biopsy, CT-guided biopsy and drain placement, and diagnostic and basic interventional angiography), and vascular surgery (surgical management of ruptured abdominal aortic aneurysm, acute limb ischemia, and management of peripheral venous disease) [32]. When considering a broad scope of training, it is important to note that the rural surgeon should be prepared to take on various roles when in practice. These roles extend beyond that of the surgeon role and may also include being a leader in trauma care, critical care, and nutrition [34].

Furthermore, an Oregon-based study aimed at examining whether training was preparing surgical residents for rural practice compared the caseloads of the residents to that of the general surgeons practicing at local CAHs. Results from the study revealed that CAH surgeons performed a more significant proportion of endoscopies as compared to surgical residents (56.1% vs. 9.1%) [33]. The scope of practice of CAH surgeons in Oregon identified from a 2012-2013 database the following procedures: endoscopy (56.1%), hernia (8.8%), cholecystectomy (6.4%), skin/soft tissue (5.4%), small/large bowel (4%), breast (3.1%), appendix (2.4%), other abdominal (2%), rectal/anal (1.6%), and esophagus/stomach (1%) [33]. To further support this idea, a 2015 cross-sectional study examining the types of procedures performed in both urban and rural hospitals in 24 states revealed that obstetric-gynecologic and orthopedic procedures were far more commonly performed by general surgeons in rural areas as compared to urban surgeons [35]. Thus, these results support the idea that more diverse caseloads during training may be beneficial for producing competent rural surgeons. In addition, the establishment of a rural track in surgical training may also aid in increasing a higher caseload for residents [35-37]. In fact, a 2018 survey revealed that 44/261 ACGME surgical residency programs identified that they had a rural surgical track or were willing to accommodate rural surgical residents [37]. By increasing the case volume of residents, this may mitigate the concern of the anticipated growth in the general surgeon to population ratio, as a sufficient case volume may play a role in training competent residents [4].

Market-Based Approach (Incentivization/Lifestyle/Autonomy/Opportunity) 

When considering the vast amount of debt that many medical students accumulate throughout their academic careers, financial incentivization has often been noted as means of attracting providers to rural areas. This often involves providing loan forgiveness in exchange for newly graduated physicians to train or work in rural areas for an established time frame [38]. Other studies have suggested a need for expansion in the existing National Health Service Corps to include all healthcare providers, including general surgeons, while also considering expansion of economic hardship grants in efforts to cast a wider net in terms of providing financial aid for those willing to serve in rural areas [38,39]. However, financial incentivization should not be considered as the sole driving factor for increasing the number of surgeons willing to work in rural areas. Instead, other factors, such as lifestyle, opportunity for career advancement, and autonomy, must be considered to utilize a market-based approach in sustaining rural surgeons. 

The lifestyle of a rural general surgeon clearly differs from urban or suburban surgeons. Rural general surgeons will find themselves in a smaller town, with fewer lifestyle complexities typically associated with metropolitan areas, such as higher cost of living, and rather more leisure opportunities through outdoor recreational activities and a more peaceful lifestyle [15,20]. Frohne et al. described the value in promoting the unique characteristics of rural life along with family support and outreach opportunities to increase interest in rural surgery [22]. As demonstrated by results of the study performed by Frohne et al., improving living conditions, childhood education systems, and opportunities for spouses may foster a greater attraction to rural surgical practice [22].

Beyond these lifestyle differences, rural general surgeons can enjoy the perks of greater autonomy and independence in surgical practice [15]. For surgeons interested in leadership roles, rural general surgery offers the ideal setting for quickly attaining leadership positions in the local hospital or community [15]. Many surgeons may find higher satisfaction and security with rural surgery if provided opportunities for teaching, mentoring, and learning at academic institutions [22]. This is especially important to consider for young surgeons interested in continuing to enhance and develop skills while in practice [39]. Oftentimes, rural hospitals may face obstacles when attempting to recruit surgeons due to the limited number of academic opportunities in the area [12]. In addition, the introduction of continuing medical education (CME) opportunities in rural settings may help to address concerns of CME inaccessibility to practicing rural surgeons [20]. In turn, this may provide the level of professional support that rural surgeons need and desire when practicing in more isolated rural areas. 

Another avenue to respond to the rural general surgeon market may be centered on the day-to-day practice of the surgeons. By introducing call schedules for rural surgeons, making improvements to existing call schedules, and even recruiting support staff, rural practice may become a more attractive option [22]. For surgeons who are largely procedure-based, the demand for strong support staff is necessary to consider when attempting to recruit surgeons whose practice is highly dependent on a reliable team in order to carry out an efficient and successful surgery schedule.

Discussion

This narrative review identifies several key initiatives that may aid in achieving a sustainable surgical workforce for rural areas. These strategies can be summarized into enacting improvements in three major categories: medical school, residency training, and physician market. While current efforts have shown a significant likelihood of fostering a sustainable surgical workforce in rural areas, there is no existing evidence that suggests a single-use approach may be more beneficial for achieving this goal. Instead, a multifaceted approach, which may rely more heavily on integrating a physician market-based perspective to provide an avenue to increase the retention of surgeons willing to practice in a rural area. By coupling this with new opportunities to train as rural surgeons through an established fellowship, graduating surgical residents may find this as a means of developing a unique skill set while taking advantage of the attributes unique to practicing in a rural area [18,20]. Otherwise, expanding the breadth of residency training for general surgeons to include more experience in performing specialty procedures in the fields of obstetrics/gynecology, orthopedics, and cardiac and vascular surgery may be key in producing competent surgeons who are ready to perform the diverse surgical needs of rural residents following residency graduation [31]. 

Broadly, improvements to the perceived general surgery lifestyle and expansion of mentorship opportunities may help in increasing students’ interest in a surgical career at the college or medical school level [40]. These ideas go hand in hand with responding to the surgical shortage through a market-based approach. In other words, efforts must be aimed at identifying and delivering desirable incentives for surgeons willing to practice in a rural area. This approach is supported by the idea that while simply expanding the surgical workforce may help to address concerns related to the overall surgeon shortage, specific efforts should focus on ways to improve a balanced distribution of providers in rural areas [40]. Furthermore, the incentives that influence the balance among surgical specialization, general surgery, and urban versus rural practice must be changed in a way that responds to these trends appropriately [40]. Whether it involves financial or lifestyle incentivations, future research should be done in this area to identify specific aspects that may appeal to surgeons. 

Additional considerations should be made in regard to policy changes at the legislative and general medical education levels. As of late 2020, bipartisan congressional leaders took action by adding 1,000 new Medicare-supported GME positions for rural hospitals [41]. More recently, the Resident Physician Shortage Reduction Act of 2021 was introduced to the Congress in March 2021. If passed, this bill would increase the number of residency training positions to 14,000 over seven years (2,000 positions per year) [42]. While these valuable efforts are currently in progress, continued support and advocacy in this area must be employed to guarantee that the growing physician shortage is addressed promptly. 

Study limitations

The limitations of this study include the following: potential bias related to the nature of narrative reviews, narrow literature search strategy, insufficient data to inform the status of available ancillary medical staff and hospital resources to sustainably support rural surgery, and limited available data regarding the actual number of performed specialty procedures, surgical complication rates, and related malpractice cases in rural settings. Additional limitations relevant to the content of this narrative review involve a lack of available data that consider any potential bias or stigmatization of the practice of rural medicine that may be introduced to students attending urban and suburban medical schools. Future studies should focus on utilizing additional databases and expanding on the breadth of rural surgery practice and training as additional data become available.

## Conclusions

With the anticipated shortage of surgeons in both rural and urban areas, this review highlights the importance of enacting immediate measures to address the concern. This review has accomplished the initial objectives of gaining a better understanding of the current state of access to surgical care in rural communities and utilizing this knowledge to provide recommendations to readily attain a sustainable number of rural surgeons. With each approach suggesting ways to address the contributory issues to the surgeon shortage, this review reveals a new avenue of integrating valuable aspects from each approach, rather than relying on a single approach. In particular, enhancing the overall pipeline of medical training to attending status may prove to be more beneficial for achieving this goal. Ultimately, this may be accomplished by introducing additional rural surgical mentorship opportunities for medical students, developing a rural surgery fellowship, and incorporating a market-based response that will correspond to attractive incentives that help to retain a sustainable number of surgeons working in rural areas.
